# Annotations

**Published:** 1892-12-10

**Authors:** 


### Annotations.

**Hygiene and Caligraphy.**

Most of us remember how carefully in our school day we were taught to hold a pen and sit at a desk, and bring our writing to the required angle of grace. Most of us know how far we have fallen off from the instruction we then received, and our friends and correspondents will in all probability confirm our own suspicion that our caligraphy would break a writing master's heart. Who, indeed, employs that carefully-taught sloping caligraphy except a few middle-aged folks who rarely take a pen in hand? For the rest of us, nature, and the necessity for writing quickly have undone the effect of training. For one person who keeps the elbow close to the side and the penholder pointing to the right shoulder, a hundred turn the pen out and leave the elbow loose. The position is easier, and, speed for speed, the result is more legible. The writing produced is, at its best, less graceful than the best specimens of the angular style which was once universally taught, but, speaking generally, it is more easily deciphered, and, after all, writing is meant to be read, not to be admired as a work of art. It is said, too, that in growing children the cramped position enforced in angular writing tended to induce spinal curvature, and it is certain that to read sloping writing involves a greater strain on the sight than the deciphering of vertical caligraphy, as the lines tend to run together to the hasty eye. It is, in fact, as if books were printed in italics instead of Roman type; in which case we would be as badly off as that German savant, who, when a book came out on a subject in which he was interested printed in the Gothic type to which the Teuton clings, said that much as he longed to read the volume, he preferred to wait for a French translation. Many of us on receiving letters written in a hand more ornamental than clear might similarly ask, for the sake of our eyesight, to have them copied by a type-writer. It is possible, indeed, that the time may come when the type-writer and not the pen will be taught in schools, and the graphologists who earn a modest living by telling character from hand-writing may find their occupation gone; but in the meantime it is a step in the right direction that in Government examinations candidates are told to make their writing as upright as possible, and one cannot but perceive that the whole tendency of the age is away from the elaborate Italian style which once wasted our space and tried our eyes.

**Mirth in a Model Lodging House.**

The Corporation of Glasgow is the proprietor of a number of model lodging-houses. Finding that it had some money at its disposal; finding, too, that there was some difficulty in housing the poorest of the city's poor, it acquired land and built lodging-houses which, for accommodation and cheapness, really are models to other communities. Glasgow has been held up before now, in both hemispheres, as possessing an unexampled municipality, and in this matter of public lodging-houses alone it has proved itself worthy of renown. For it was not enough, in the opinion of provost, baillies, and councillors of St. Mungo's City to build the best homes possible for the struggling and casual poor; they sought to brighten the gloom of lives which—often, it may be, through their own fault, but not, for that, the less pitiable—are dull and gloomy in the extreme. Therefore they have organised a series of Saturday evening entertainments in the lodging-houses, at the first of which the Lord Provost presided, and a Bailie showed scenes of foreign travel by means of lime-light and a magic-lantern. Similar entertainments have followed in all the lodging-houses possessed by the municipality, and hitherto the rule has been that two members of the municipality should be present at each, one to preside and one to help to amuse the inmates. The services of outsiders, singers and reciters, are not despised, but the primary idea is that some members of the corporation—the landlord—should be present to show an interest in its tenants. So far the idea has succeeded admirably. The hall of the lodging-houses has been crowded, the audience attentive, and the entertainment appreciated. At the end of the first concert one of the lodgers rose and expressed on behalf of his fellows their appreciation of it, and of the kind thought which prompted it. This is the important point. The occupants of these lodging-houses are sometimes men who have fallen, sometimes men who have yet to rise, but the majority dwell on a dead level of despondency in which there is no past or future, nothing but casual toil for scanty pay. If some gleam of kindness, some touch of nature's kinship comes to them from outside, they become at once more contented with their lot and raised above it. They are more likely to feel themselves not casual "dossers," but citizens; they no longer dwell apart from those richer than themselves in isolated envy; they can work loyally for those who regard them from the level ground of common humanity. In conclusion we would say that while much is talked of everywhere about the welfare of the poor, a good deal has been done in the rough, kindly city of the West without much talk about it. In this matter of building and owning clean and well-kept lodging-houses, and interesting yourselves in the occupants of them, Lord Mayor, Aldermen, and Councillors of the City of London, please copy.

**See-saw.**

Mr. Rudyard Kipling has been in America, and, like other considerable persons, he has published his "impressions" on his return. Mr. Kipling thinks that the Americans who dwell "down East" are too lazy to be prosperous, and that those who inhabit the more familiar regions near the "hub of the Universe" are too nervous and fussy to be happy or well. The ink has hardly dried up on Mr. Kipling's "copy" before Miss Elizabeth L. Banks steps into the arena. She is a valiant American woman, and does not estimate Mr. Kipling at too high a price. Miss Banks thinks the "down East" Americans have the right to be lazy if they choose, seeing that their fathers and grandfathers led magnificently strenuous lives. She half admits, however, that the "nervous" Americans are not to be so easily defended. Both she and Mr. Kipling agree that "a nation does not progress wholly upon its 'brain-pan.'" "Perhaps," says Miss Banks, "too much nervous energy is debilitating in the long run. People are very apt to die with St. Vitus' dance or something of that kind if they hurry so." Physiology is evidently looking up as a factor in life when people of the Banks and Kipling order are taking the matter so soberly to heart. It is pretty clear that the modern man must have a little faith in the "gospel of rush." If he cannot put his best foot foremost with a will he will be left behind. But then there is another gospel which, though it is the reverse, yet also belongs to the same coin as the "gospel of rush," and that is the "gospel of repose." An exclusive devotion to either of these gospels is fatal. What is wanted is a judicious combination. The true physiological, and therefore the true practical life is a kind of game of see-saw. We must be alternately "up and down": up at work and then down at rest, at pleasure, at play. The nervous American likes to be always up; and so he "dies of St. Vitus' dance or something of that kind." The stolid Englishman, on the whole, manages these things better. His pride does not permit him to be too fussy and nervous; he has a contempt for that sort of thing. The Englishman is right and the American wrong. We shall wear out our transatlantic brethren yet, if they do not learn the art of resting with dignity as well as that of working with fury.

